# Changes in Veterans Health Administration Emergency Department Visits During Two Years of COVID-19

**DOI:** 10.5811/westjem.18714

**Published:** 2025-06-20

**Authors:** Justine Seidenfeld, Aaron Dalton, Anita A. Vashi

**Affiliations:** * Durham Veterans Affairs Health Care System, Center of Innovation to Accelerate Discovery and Practice Transformation (ADAPT), Durham, North Carolina; † Durham Veterans Affairs Health Care System, Department of Emergency Medicine, Durham, North Carolina; ‡ Duke University School of Medicine, Department of Emergency Medicine, Durham, North Carolina; § Palo Alto Veterans Affairs Health Care System, Center for Innovation to Implementation, Palo Alto, California; || Stanford University School of Medicine, Department of Emergency Medicine (Affiliated), Stanford, California; # University of California San Francisco School of Medicine, Department of Emergency Medicine, San Francisco, California

## Abstract

**Introduction:**

To better understand the impact of the COVID-19 pandemic on emergency department (ED) utilization, we examined two years of Veterans Health Administration (VHA) ED visits. Emergent and non-emergent ED visits were examined separately to understand the impact of systems-level changes in healthcare delivery.

**Methods:**

In this retrospective, observational cohort study we examined ED visits in 111 EDs within the VHA from March 2018–February 2022. Primary outcome was the count of emergent and non-emergent ED visits, using incident rate ratios (IRR) with 95% confidence intervals (CI) to examine ED visits during the first two years of the COVID-19 pandemic in eight separate quarters, compared to two years of seasonally equivalent quarters before COVID-19.

**Results:**

Over the four-year period, US veterans made 8,057,011 ED visits, with 54.7% in the eight pre-COVID-19 quarters, and 45.3% in the first eight quarters during the COVID-19 pandemic. Both emergent and non-emergent visit counts decreased in each of the first eight quarters during COVID-19 when compared to their respective pre-COVID-19 baseline. The change in emergent visits ranged between −26.9% (March–May 2020; IRR 0.73, 95% CI 0.72–0.74) and −7.0% (June–August 2021; IRR 0.93, 95% CI 0.92–0.94). The change in non-emergent visits ranged between −33.0% (March–May 2020; IRR 0.67, 95% CI 0.67–0.67) and −5.7% (June–August 2021; IRR 0.94, 95% CI 0.94–0.95). After the first six months of the pandemic, emergent ED visits had a sustained greater decrease compared to non-emergent visits.

**Conclusion:**

As of 2022, ED visits had not returned to pre-pandemic baselines, and our results suggest that emergent visits have sustained a greater decrease even in the second year of the pandemic compared to their respective, seasonally equivalent pre-pandemic quarters from March 2018–February 2020. The finding that emergent visits decreased more than non-emergent is notable given that system-level changes in care delivery, particularly a shift toward use of telehealth, would be expected to have a greater impact on non-emergent care. More work is needed to understand whether acute care is being forgone altogether, as well as the subsequent impact.

## INTRODUCTION

During the COVID-19 pandemic, disruptions including lockdowns, fears of virus transmission, and restrictions on non-emergent medical services, broadly impacted healthcare delivery in both the outpatient and inpatient settings.^1^ As emergency department (ED) utilization is intricately linked to care in those settings, this created an environment in which EDs became a crucial point of access to emergent and non-emergent healthcare for many, including veterans of the United States military.^2^ Similar to community settings, previous work in the Veterans Health Administration (VHA) suggested that even with a large shift in outpatient care from in-person to telehealth settings during the first year of the pandemic, overall numbers of scheduled outpatient appointments still declined.^3^,^4^ Given this decline, it is important to understand how known disruptions in both acute and chronic care during the COVID-19 pandemic impacted ED utilization for both emergent and non-emergent care.

Previous work looking at ED visits in the community has demonstrated decreases in specific emergent diagnoses (eg, stroke, acute myocardial infarction), largely during the initial year of the pandemic.^5^–^8^ Other studies from the early phase of the pandemic also demonstrated fewer admissions from the ED.^9^ More recent work looking at the impact of COVID-19 on ED outcomes beyond the first year of the pandemic has focused on ED boarding and in-hospital mortality but has not examined the types of ED visits seen during these periods.^10^–^12^

However, as COVID-19 cases continue to fluctuate, a better understanding of comprehensive trends in ED utilization for emergent and non-emergent care is needed beyond specific types of ED presentations. This approach can better represent the impacts of system-level changes in healthcare delivery outside the ED during the pandemic. In this study we aimed to add to this body of literature by examining changes in ED visit rates for emergent vs non-emergent ED visits in a national health care system over two years. With data spanning beyond the pandemic’s initial phases, this study will contribute to a better understanding of the long-term impacts of changes in healthcare delivery in both outpatient and inpatient settings on ED utilization during the COVID-19 pandemic.

## METHODS

This was a retrospective, observational, cohort study of ED visits within the VHA from March 2018– February 2022. The VHA represents the largest integrated health system in the US, with 111 EDs, and provides services to more than nine million enrolled veterans. This study was approved by the Stanford University Institutional Review Board (IRB) and by the VA Palo Alto Research & Development Committee. This study followed the Strengthening the Reporting of Observational Studies in Epidemiology (STROBE) reporting guidelines (Supplemental Materials). Additionally, while our study was based on administrative data, we adhered to several established criteria to enhance methodological rigor in medical record review studies in ED research.^13^ These included case selection criteria, variable definition, medical record identified, sampling method, and IRB approval.

The ED encounter and sociodemographic data were retrieved from the VA Corporate Data Warehouse. The overall study sample included all VA ED encounters by veterans who were ≥18 years of age. We defined ED encounter diagnoses for emergency care-sensitive conditions (ECSC) by the International Classification of Diseases, 10^th^ Rev, Clinical Modification (ICD-10-CM) code. The ECSCs, for which timely access to emergency care impacts morbidity and mortality, were defined previously by a multidisciplinary review panel with emergency medicine, primary care, and hospitalist experts, using a modified Delphi method. The ECSCs encompass 51 condition groups with associated ICD-10-CM codes, such as sepsis or systemic inflammatory response syndrome, chronic obstructive pulmonary disease, pneumonia, asthma, and heart failure, among others.^14^ We excluded from this analysis ED visits clearly attributable to COVID-19 illness (those with an ED encounter diagnosis of U07.1 and U07.2). We collected baseline patient characteristics including age, sex, race, ethnicity, VA priority group, driving distance to closest VA ED, residential location, marital status, and Elixhauser Comorbidity Index score.^15^ Veterans Administration priority groups, which are used to determine the costs a veteran has to pay toward their care, are calculated using veterans’ military service history, service-connected disabilities, and other factors.

The primary outcome was the count of ECSC and non-ECSC ED visits. We generated incident rate ratios (IRR) with 95% confidence intervals (CI) to examine changes in ECSC and non-ECSC ED visits across eight quarters (three-month intervals) spanning the initial two years of the COVID-19 pandemic. For each quarter during those two years, the IRR presented is a ratio that compares the daily rate of ECSC or non-ECSC ED visits during that single COVID-19 quarter to the average of two daily rates observed in corresponding quarters (meaning the same calendar months) before the pandemic in 2018 and 2019. This approach provided a meaningful comparison, allowing us to gauge the impact of COVID-19 on ED visit rates while also accounting for seasonal variations in the pre-COVID-19 years, and is consistent with other pre/post-COVID-19 ED utilization comparisons in the literature.^5^,^16^–^17^ Pre-COVID-19 periods spanned March 2018–February 2020, and during-COVID-19 periods were March 2020–February 2022. For example, March–May 2020 (COVID-19 quarter 1) is compared to the average of March–May 2018 and March–May 2019. Similarly, March–May 2021 (COVID-19 quarter 5) is compared to the average of March–May 2018 and March–May 2019. June–August 2021 (COVID-19 quarter 6) is compared to the average of June–August 2018 and June–August 2019. Encounter-level demographic characteristics are described using summary statistics. We compared both ECSC and non-ECSC visits between pre-COVID-19 and during-COVID-19 periods with standardized mean differences. For all analyses, significance was set at *P*< 0.05. We conducted all analyses in R version 4.1.2 (R Foundation for Statistical Computing, Vienna, Austria).

## RESULTS

Over the four-year period, US veterans made 8,057,011 ED visits, with 4,410,123 (54.7%) in the eight pre-COVID-19 quarters, and 3,646,888 (45.3%) in the eight quarters during the COVID-19 pandemic. The overall enrollee population increased by approximately 1.1% over this time period, with counts as follows: fiscal year (FY) 2018–9,898,266; FY 2019–9,929,810; FY 2020–9,888,475; and FY 2021–10,010,358. For both ECSC and non-ECSC visits, there were no significant imbalances in patient characterisitics when comparing the pre-COVID-19 and during-COVID-19 periods. When comparing ECSC to non-ECSC ED visits, patients were older, more likely to be male, and had higher mean Elixhauser Comorbidity Index scores (Table).

Both ECSC and non-ECSC visits counts decreased throughout all eight quarters during COVID-19 compared to their pre-COVID-19 equivalent quarters (Figure); total visit count trends are presented in the Supplemental Materials. Of note, there was a steady increase in the total number of VA ED visits in the four years preceding the COVID-19 pandemic, with counts as follows: FY 2016–2,105,766; FY 2017–2,137,716; FY 2018– 2,209,497; and FY 2019– 2,215,048. This trend further underscores the subsequent changes in ED visit counts during the COVID-19 pandemic period.

In the first quarter of the pandemic, non-ECSC visits had a greater decrease than ECSC visits. When comparing March-May 2020 (Quarter 1) to the pre-pandemic baseline quarters of March-May 2018 and 2019, non-ECSCs were 33.0% lower (IRR 0.67, 95% CI 0.67–0.67) and ECSCs were 26.9% lower (IRR 0.73, 95% CI 0.72–0.74). This gap narrowed in June-August 2020 (Q2); non-ECSCs were 18.8% lower (IRR 0.81, 95% CI 0.81–0.82), and ECSCs were 17% lower (IRR 0.83, 95% CI 0.82–0.84) when compared to their pre-pandemic baseline visit counts in June–August 2018 and 2019. After that, ECSC visits had a greater relative decrease than non-ECSC visits in each of the six following quarters (covering September 2020–February 2022) when compared to their pre-pandemic baseline.

As expected, the greatest decreases in both ECSC and non-ECSC visits were from March-May 2020 (Q1), although ECSC visits had a similar decrease of −26.7% (IRR 0.73, 95% CI 0.73–0.74) between December 2020–Feb 2021 (Q4). Both ECSC and non-ECSC visits returned closest to their pre-pandemic baseline in June–August 2021 (Q6), with a −7% change in ECSC visits (IRR 0.93, 95% CI 0.92–0.94) and a −5.7% change in non-ECSC visits (IRR 0.94, 95% CI 0.94–0.95). Both ECSCs and non-ECSCs again then declined further from their pre-pandemic baselines in the subsequent two quarters from September 2021–February 2022 (Q7–Q8).

## DISCUSSION

Examining two years of ED visits during the COVID-19 pandemic in the VHA, we demonstrate that as of 2022, neither emergent nor non-emergent ED visits returned to their pre-pandemic baselines. Beyond the initial six months of the pandemic, emergent visit rates were still lower compared to non-emergent visits. Our results are consistent with a previous study through August 2022, demonstrating that ED visits did not fully recover in the second year of the pandemic compared to pre-pandemic years (while ED crowding increased), and similarly show fluctuations with peak decreases in visits during winter seasons.^18^ However, our results suggest different trends in emergent ED visits in the second year of the pandemic.

Oskvarek et al examined trends in illness severity based on Emergency Severity Index (ESI) level and critical care billing, but they did this at a yearly level, demonstrating an overall increased proportion of ED visits for ESI levels 1 and 2 in 2022 (18.9%) vs 2019 (17.9%). In our study, we characterized severity based on emergency care sensitive diagnoses and conversely demonstrated that emergent visit rates have decreased even more than non-emergent visits. This difference in results may be due to the approach to characterizing emergent vs non-emergent visits. An approach based on final ED visit discharge diagnoses may better capture the broader continuum of emergent ED services compared to an initial triage-based assessment. Additionally, we elected to exclude ED visits specifically for COVID-19 in our analysis to better understand how COVID-19-driven changes in outpatient care impacted routine “non-COVID-19” ED care, which may have accounted for more “high-acuity” visits in their study.

Overall, these findings demonstrating even greater sustained decreases in emergent over non-emergent ED visits are notable. Considering the impact of increased use of telehealth for outpatient care,^3^ we might have expected a relatively greater decrease in non-emergent ED visits, as these may be more amenable to virtual care. However, veterans’ access to and acceptability of telehealth varies,^19^ and more work is needed to understand whether both ECSC and non-ECSC visits have similarly shifted to telehealth or are being forgone altogether, as well as subsequent clinical impact. Additionally, while increased use of community care EDs would also have contributed to the general decrease in ED visits, the specific impact of this shift on these emergent and non-emergent categories is unknown, and an important direction for future work.^20^ Understanding these trends is vital for assessing the potential implications on health system capacity and use of alternative sites of care.

## LIMITATIONS

We recognize that this study has several limitations. First, it is focused on a population of US military veterans, which may limit generalizability to other systems. This study did not include non-VA-delivered ED visits made by veterans and paid for by Medicare or other insurance, which may have affected the observed changes observed during COVID-19 and limit generalizability to veterans who sought ED care outside the VA system. Additionally, use of ECSCs as a method to define emergent ED visits is based on administrative data and, thus, is limited in its ability to fully capture clinical severity. The use of ICD-10 codes to define excluded COVID-19 visits also poses a risk of including visits for viral illnesses that were in fact COVID-19, potentially leading to an undercount of ED visits not directly attributed to COVID-19. However, we selected this approach to ensure that we did not exclude other viral respiratory conditions. Finally, as this was an administrative study, we were limited in our ability to suggest causal reasons for declines in either emergent or non-emergent ED visits. Nevertheless, this work provides valuable insights and can suggest directions for further investigation.

## CONCLUSION

While COVID-19 is no longer deemed a public health emergency, it remains imperative to assess whether the recurring pattern of reduced ED visits observed during the second year of the pandemic persists. These changes in care-seeking behavior may be due to subsequent infection waves, as well as more persistent changes in healthcare delivery and health systems outside the ED setting.

## Supplementary Information





## Figures and Tables

**Figure:**
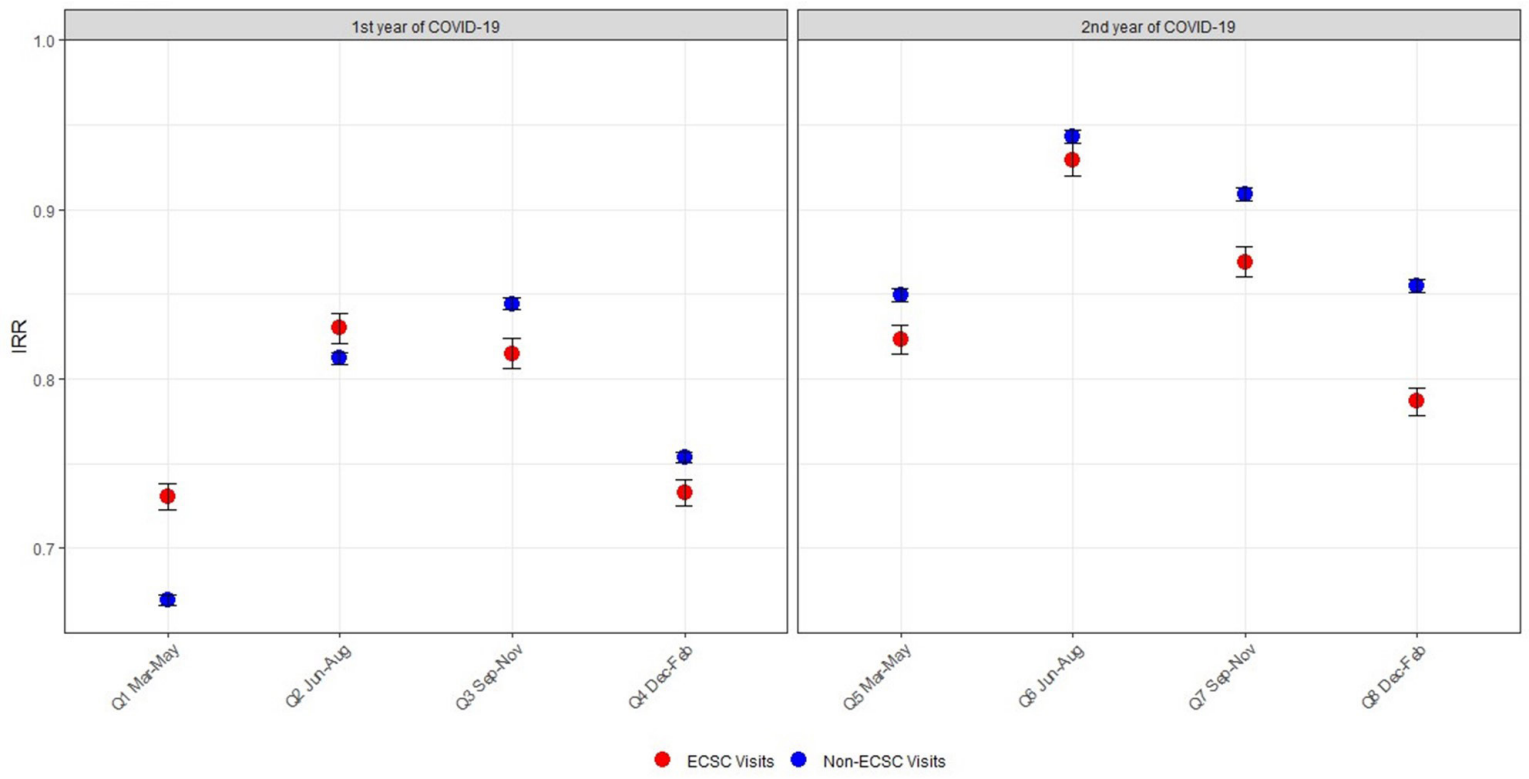
Incident rate ratios and 95% confidence intervals of emergency department visits during COVID-19 compared to pre-pandemic years by quarter. Ratio rates <1.0 indicate fewer ED visits in the COVID-19 quarter. *ECSC*, emergency care-sensitive condition; *IRR*, incident rate ratio.

**Table:** Demographic and clinical characteristics of Veterans Health Administration patients by emergency department visit type.

	ECSC visits (n=1,136,248)			Non-ECSC visits (n=6,920,763)		
	
	Pre-COVID-19^a^ (n=626,801)	During COVID-19^a^ (n=509,447)	Standardized mean difference for ECSC visits^b^	Pre-COVID-19^a^ (n=3,783,322)	During COVID-19^a^ (n=3,137,441)	Standardized mean difference for non-ECSC visits^b^
Age, mean (SD)	67.80 (13.5)	68.22 (13.6)	0.031	60.09 (16.2)	60.29 (16.3)	0.012
Age, years, n (%)			0.05			0.045
18–44	41,837 (6.7)	35,845 (7.0)		746,569 (19.7)	647,837 (20.6)	
45–64	173,028 (27.6)	130,657 (25.6)		1,347,560 (35.6)	1,058,425 (33.7)	
65–84	347,342 (55.4)	292,642 (57.4)		1,480,791 (39.1)	1,268,603 (40.4)	
85+	64,594 (10.3)	50,303 (9.9)		208402 (5.5)	162,576 (5.2)	
Sex, n (%)			0.015			0.013
Male	590,550 (94.2)	478,209 (93.9)		3,360,542 (88.8)	2,773,861 (88.4)	
Race, n (%)			0.046			0.044
American Indian or Alaska Native	4,852 (0.8)	4,073 (0.8)		31,232 (0.8)	26,627 (0.8)	
Asian	3,194 (0.5)	2,882 (0.6)		29,881 (0.8)	28,246 (0.9)	
Black	146,660 (23.4)	126,138 (24.8)		1,082,916 (28.6)	928,593 (29.6)	
Native Hawaiian or other Pacific Islander	4,308 (0.7)	3,749 (0.7)		29,968 (0.8)	26,699 (0.9)	
White	437,674 (69.8)	345,284 (67.8)		2,407,051 (63.6)	1,937,413 (61.8)	
Ethnicity, n (%)			0.021			0.036
Not Hispanic or Latino	574,833 (91.7)	463,393 (91.2)		3,395,850 (89.8)	2,783,474 (88.7)	
VA priority group, n (%)			0.073			0.09
1 and 4: high disability	289,712 (46.2)	242,313 (47.6)		1,855,833 (49.1)	1,637,210 (52.2)	
2, 3, and 6: low/moderate disability	107,612 (17.2)	89,556 (17.6)		713,188 (18.9)	571,864 (18.2)	
5: Low income	169,001 (27.0)	122,410 (24.0)		852,578 (22.5)	602,678 (19.2)	
7–8: non-disabled; copayment required	60,445 (9.6)	55,164 (10.8)		361,540 (9.6)	325,638 (10.4)	
VA ED driving distance miles, n (%)			0.068			0.06
0–10 miles	209,599 (33.5)	142,526 (35.5)		1,240,286 (32.8)	843,541 (34.3)	
10–20 miles	148,020 (23.6)	96,521 (24.0)		935,931 (24.8)	620,026 (25.2)	
20–30 miles	80,262 (12.8)	51,833 (12.9)		512,430 (13.6)	340,367 (13.8)	
30–40 miles	51,555 (8.2)	33,063 (8.2)		315,869 (8.4)	206,196 (8.4)	
40–50 miles	33,665 (5.4)	20,097 (5.0)		190,582 (5.0)	117,478 (4.8)	
50–60 miles	24,704 (3.9)	14,798 (3.7)		137,017 (3.6)	81,496 (3.3)	
>60 miles	78,074 (12.5)	42,727 (10.6)		444,804 (11.8)	250,040 (10.2)	
Residential location, n (%)			0.051			0.046
Urban	458,149 (73.1)	381,260 (74.8)		2,920,960 (77.2)	2,471,039 (78.8)	
Rural	154,794 (24.7)	115,813 (22.7)		796,698 (21.1)	607,019 (19.3)	
Highly rural	13,240 (2.1)	12,103 (2.4)		61,807 (1.6)	57,281 (1.8)	
Unhoused, n (%)	72,501 (11.6)	56,116 (11.0)	0.017	520,066 (13.7)	398,953 (12.7)	0.03
Marital status, n (%)			0.021			0.03
Married	270,820 (43.2)	221,374 (43.5)		1,552,476 (41.0)	1,281,284 (40.8)	
Not married	353,368 (56.4)	285,172 (56.0)		2,212,396 (58.5)	1,833,501 (58.4)	
Elixhauser Comorbidity Index score, mean (SD)	6.33 (3.42)	6.23 (3.34)	0.028	3.97 (3.18)	3.76 (3.00)	0.066
Elixhauser Comorbidity Index score, n (%)			0.02			0.039
0	8,830 (1.4)	8,319 (1.6)		384,238 (10.2)	339,031 (10.8)	
1	27,379 (4.4)	22,204 (4.4)		509,529 (13.5)	444,595 (14.2)	
2	44,142 (7.0)	36,932 (7.2)		570,993 (15.1)	489,461 (15.6)	
≥3	546,450 (87.2)	441,992 (86.8)		2,318,562 (61.3)	1,864,354 (59.4)	

Percentages in table may not add to 100% due to missing values.

a Pre-COVID-19 periods: March 2018–February 2020; during-COVID-19 periods: March 2020 –February 2022.

b SMD ≤0.2 indicates variable balance between populations.

c VA enrollment priority group is calculated using military service history, service-connected disability, income, Medicaid qualification, and other VA benefits, and determines the amount of co-payment required.

*ECSC*, emergency care-sensitive condition; *ED*, Emergency Department; *SMD*, standardized mean difference; *VA*, Veterans Affairs.
