# Supplementary figures and images for: Changes in Veterans Health Administration Emergency Department Visits During Two Years of COVID-19
Source: West J Emerg Med. 2025 Jun 20;26(4):869–75. doi: 10.5811/westjem.18714 (PMC12342404; doi:10.5811/westjem.18714)

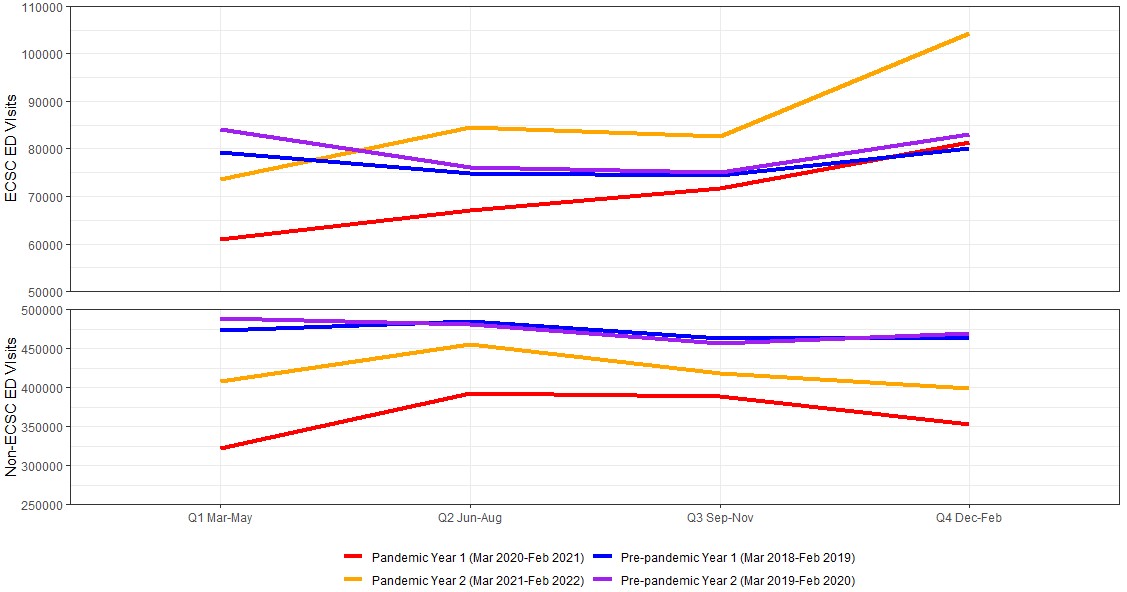

Supplement: Supplementary file 1 [file wjem-26-869-s001.jpg]
